# Modelo de inteligencia artificial para la detección temprana de diabetes

**DOI:** 10.7705/biomedica.7147

**Published:** 2023-12-29

**Authors:** William Hoyos, Kenia Hoyos, Rander Ruiz-Pérez

**Affiliations:** 1 Grupo de Investigación en Ingeniería Sostenible e Inteligente, Universidad Cooperativa de Colombia, Montería, Colombia Universidad Cooperativa de Colombia Universidad Cooperativa de Colombia Montería Colombia; 2 Grupo de Investigaciones Microbiológicas y Biomédicas de Córdoba, Universidad de Córdoba, Montería, Colombia Universidad de Córdoba Universidad de Córdoba Montería Colombia; 3 Laboratorio Clínico, Clínica Salud Social, Sincelejo, Colombia Clínica Salud Social Sincelejo Colombia; 4 Grupo de Investigación Interdisciplinario del Bajo Cauca y Sur de Córdoba, Universidad de Antioquia, Medellín, Colombia Universidad de Antioquia Universidad de Antioquia Medellín Colombia

**Keywords:** diabetes-diagnóstico, predicción, factores de riesgo, sistema de apoyo a la decisión clínica, inteligencia artificial, diabetes/diagnosis, forecasting, risk factors, clinical decision support system, artificial intelligence

## Abstract

**Introducción.:**

La diabetes es una enfermedad crónica que se caracteriza por el aumento de la concentración de la glucosa en sangre. Puede generar complicaciones que afectan la calidad de vida y aumentan los costos de la atención en salud. En los últimos años, las tasas de prevalencia y mortalidad han aumentado en todo el mundo. El desarrollo de modelos con gran desempeño predictivo puede ayudar en la identificación temprana de la enfermedad.

**Objetivo.:**

Desarrollar un modelo basado en la inteligencia artificial para apoyar la toma de decisiones clínicas en la detección temprana de la diabetes.

**Materiales y métodos.:**

Se llevó a cabo un estudio de corte transversal, utilizando un conjunto de datos que incluía edad, signos y síntomas de pacientes con diabetes y de individuos sanos. Se utilizaron técnicas de preprocesamiento para los datos. Posteriormente, se construyó el modelo basado en mapas cognitivos difusos. El rendimiento se evaluó mediante tres parámetros: exactitud, especificidad y sensibilidad.

**Resultados.:**

El modelo desarrollado obtuvo un excelente desempeño predictivo, con una exactitud del 95 %. Además, permitió identificar el comportamiento de las variables involucradas usando iteraciones simuladas, lo que proporcionó información valiosa sobre la dinámica de los factores de riesgo asociados con la diabetes.

**Conclusiones.:**

Los mapas cognitivos difusos demostraron ser de gran valor para la identificación temprana de la enfermedad y en la toma de decisiones clínicas. Los resultados sugieren el potencial de estos enfoques en aplicaciones clínicas relacionadas con la diabetes y respaldan su utilidad en la práctica médica para mejorar los resultados de los pacientes.

## Abstract (en)

**Introduction.:**

Diabetes is a chronic disease characterized by a high blood glucose level. It can lead to complications that affect the quality of life and increase the costs of healthcare. In recent years, prevalence and mortality rates have increased worldwide. The development of models with high predictive performance can help in the early identification of the disease.

**Objective.:**

To develope a model based on artificial intelligence to support clinical decision-making in the early detection of diabetes.

**Materials and methods.:**

We conducted a cross-sectional study, using a dataset that contained age, signs, and symptoms of patients with diabetes and of healthy individuals. Pre-processing techniques were applied to the data. Subsequently, we built the model based on fuzzy cognitive maps. Performance was evaluated with three metrics: accuracy, specificity, and sensitivity.

**Results.:**

The developed model obtained an excellent predictive performance with an accuracy of 95%. In addition, it allowed to identify the behavior of the variables involved using simulated iterations, which provided valuable information about the dynamics of the risk factors associated with diabetes.

**Conclusions.:**

Fuzzy cognitive maps demonstrated a high value for the early identification of the disease and in clinical decision-making. The results suggest the potential of these approaches in clinical applications related to diabetes and support their usefulness in medical practice to improve patient outcomes.

La diabetes es una enfermedad crónica no transmisible que genera grandes pérdidas anuales, humanas y económicas, a nivel mundial.

La presencia de esta enfermedad reduce la calidad de vida, disminuye la productividad del paciente, aumenta la tasa de mortalidad, aumenta los costos en los sistemas de salud por la demanda en la atención y el diagnóstico, y los altos precios de los tratamientos ^1^. Por lo tanto, son de gran importancia la prevención y el diagnóstico oportuno de la diabetes. Con el avance de la tecnología, se han desarrollado sistemas computacionales que permiten apoyar la toma de decisiones médicas, los cuales combinan técnicas de inteligencia artificial con información clínica para contribuir a mejorar la atención de los pacientes ^2^.

En diversos trabajos se han utilizado técnicas de inteligencia artificial para la predicción de la diabetes, como las máquinas de soporte vectorial ^3^, bosques aleatorios ^4^, regresión logística ^5^, XGBoost ^6^ y redes neuronales artificiales ^7^. Sin embargo, son pocos los estudios en que se han utilizado los mapas cognitivos difusos *(Fuzzy Cognitive Maps,* FCM) para predecirla. Por ejemplo, Giles *et al.* demostraron el potencial de los mapas cognitivos difusos para extraer e integrar perspectivas de conocimientos de los factores determinantes de la diabetes ^8^. Alam los usó para analizar la relación existente entre los síntomas de la diabetes y los factores de riesgo ^9^. Bhatia y Kumar desarrollaron un sistema basado en dichos mapas para predecir la diabetes, empleando síntomas y factores de riesgo ^10^. Hoyos *et al.* propusieron un enfoque para el desarrollo de modelos prescriptivos denominado PRV-FCM (PRescriptiVe-FCM) implementando el uso de los mapas cognitivos difusos optimizados con un algoritmo genético ^11^.

A pesar de que se han desarrollado investigaciones usando estos mapas cognitivos para la diabetes, no se evalúa el desempeño predictivo de los modelos en conjuntos de datos, sino que se utilizan datos simulados limitados. Además, en varios estudios no se han usado simulaciones computacionales para evaluar el comportamiento de las variables a lo largo del tiempo.

Con base en este contexto, el objetivo de este estudio fue desarrollar un sistema de apoyo a la toma de decisiones clínicas basado en mapas cognitivos difusos, como herramienta para el diagnóstico oportuno de la diabetes. Específicamente, la presente investigación aporta varias contribuciones al desarrollo actual de tal sistema:

- un modelo basado en mapas cognitivos difusos para predecir la diabetes y evaluar el comportamiento de las variables involucradas, y
- una comparación cualitativa con otros trabajos reportados en la literatura científica.

## Materiales y métodos

En esta sección, se presenta la metodología para cumplir el objetivo principal de esta investigación. Primero, se describe el conjunto de datos usado y, posteriormente, las etapas para la construcción del modelo de inteligencia artificial para la predicción de la diabetes. En la figura 1, se aprecia el esquema general del proceso, desde la recolección de los datos hasta la evaluación del modelo predictivo propuesto.

**Figura 1:**
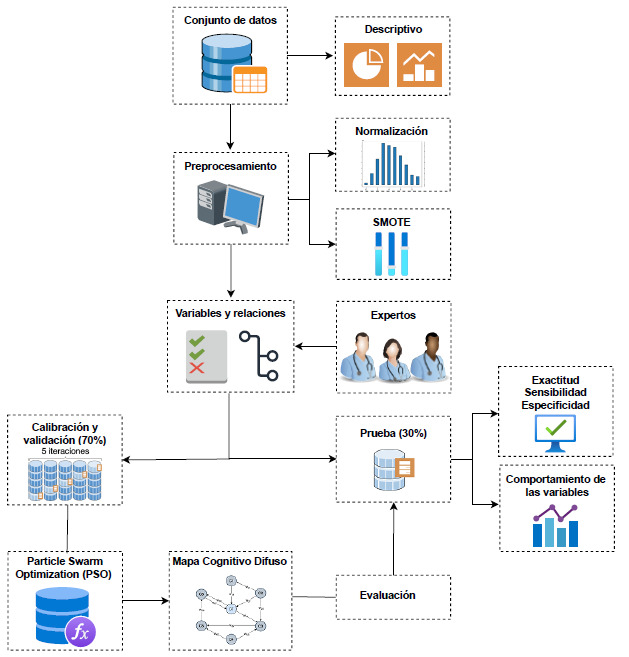
Marco secuencial del proceso de investigación

### Tipo de estudio

Se trata de un estudio cuantitativo de corte transversa!, con datos recolectados en un solo punto de! tiempo usando encuestas. No se resaltan nuevos datos de los participantes, sino que se analizaron los registros de un conjunto de datos ya existente.

### Conjunto de datos

Se utilizó el conjunto de datos de acceso libre del *Sylhet Diabetic Hospital* (Bangladés), el cual contiene los signos y síntomas de 520 individuos bajo supervisión médica ^12^. Este conjunto de datos está disponible para su descarga desde internet. La información de esta base de datos fue obtenida mediante encuesta que se les hizo a 320 pacientes con diagnóstico de diabetes y a 200 individuos sin la enfermedad. En el cuadro 1 del apéndice 1, se observa la información de las 17 variables en estudio (incluida una variable de clase, que corresponde al diagnóstico de diabetes), de las cuales todas son binarias (Sí-No), excepto la edad.

### Análisis descriptivo

Se hizo un análisis descriptivo del conjunto de datos, para conocer su distribución. En el análisis de las variables cualitativas (binarias), se utilizó la distribución por frecuencias absolutas y relativas, en relación con el diagnóstico de diabetes. Para el análisis de la edad, se emplearon medidas de tendencia central y de dispersión, tales como la media y la desviación estándar, respectivamente.

### Preprocesamiento de los datos

Teniendo en cuenta que en el conjunto de datos la distribución de las clases se encontraba desequilibrada, con el 38,47 % de registros de individuos sin diabetes y el 61,53 % de registros de pacientes con diabetes, se utilizó la técnica de sobremuestreo de minorías sintéticas *(Synthetic Minority Oversampling Technique,* SMOTE) ^13^. Mediante esta técnica, se muestrearon datos de la clase inferior; así, se generaron nuevas instancias a partir de los datos minoritarios existentes y se aumentó el número de datos para la etiqueta menor, es decir, se ajustaron a 320 registros de individuos sin diabetes frente a los 320 registros de pacientes con diabetes, obteniéndose un conjunto de datos con 640 registros equilibrados entre sus clases.

También, se utilizó la técnica de normalización mín-máx para la edad, que fue la única variable numérica del conjunto de datos. Este procedimiento ayudó a mantener las variables en el mismo rango y, además, a optimizar el tiempo en el entrenamiento de los modelos. Una mayor explicación de esta técnica puede verse en el apéndice 2.

### Definición de un mapa cognitivo difuso

Los mapas cognitivos difusos son técnicas computacionales que buscan simular el razonamiento humano, en forma similar a como lo hacen los expertos o cualquier persona con conocimientos sobre un tema en particular ^8^. Para ello, se emplearon la representación gráfica de los elementos o conceptos que conforman un sistema y las relaciones entre estos ^14^. Desde su introducción por Kosko ^15^ en 1986, los mapas cognitivos difusos han venido evolucionando ^16^ y permitiendo la obtención de nuevo conocimiento en la última década ^17^. De esta forma, en la actualidad, un mapa cognitivo difuso es una poderosa herramienta para modelar sistemas con relaciones complejas ^14^. Una explicación más completa de esta técnica puede verse en el apéndice 3. Dichos mapas pueden representarse de manera gráfica (figura 1 en apéndice 4) o de manera matemática usando una matriz de números (cuadro 2 del apéndice 1).

### Calibración y validación del mapa cognitivo difuso

Para la calibración y validación, se utilizó el 70 % de los datos y, para probar el modelo, se empleó el 30 % restante ^18^. Se utilizó la técnica de validación cruzada de cinco repeticiones, cuyo propósito fue obtener el mejor modelo y sus hiperparámetros ^7^. En la figura 2 del apéndice 4, se ilustra el proceso general de la validación cruzada de cinco repeticiones.

Como se observa en el esquema, el conjunto de datos de calibración y validación (70 %) se subdividió en cinco subconjuntos, de los cuales se emplearon cuatro para la calibración y uno para la validación. Este mismo procedimiento se ejecutó sobre un subconjunto distinto del anterior, respetando la misma proporción (4:1) de datos. Luego de cinco iteraciones, se seleccionó el mejor modelo y sus hiperparámetros, con el cual se hicieron las pruebas de rendimiento, utilizando el 30 % de los datos del subconjunto de prueba. Para la calibración, se utilizaron múltiples configuraciones de parámetros, con el fin de hallar la mejor configuración en la construcción de un modelo de mapa cognitivo difuso. Estas configuraciones pueden observarse en el cuadro 3 del apéndice 1.

En el presente estudio, se adoptó una estrategia mixta: inicialmente, tres médicos internistas expertos en el manejo de la diabetes asignaron las influencias entre las variables del conjunto de datos, para luego optimizar el modelo mediante la aplicación de técnicas computacionales. En la práctica, esto permitió articular la intervención humana con uno de los algoritmos más utilizados para el diseño de dichos mapas: la optimización por enjambre de partículas *(Particle Swarm Optimization,* PSO), descrita así por Kennedy y Eberhart en 1995, por la similitud con el comportamiento de los enjambres de insectos en la naturaleza ^19^. Este es un algoritmo de búsqueda que se usó sobre el conjunto de datos para hallar el mapa cognitivo difuso que mejor describiera las relaciones entre los conceptos o variables. Esta estrategia permite la obtención de modelos de mapas cognitivos difusos óptimos para la descripción, predicción o evaluación del comportamiento de las características de un sistema en estudio ^11^. Una descripción detallada de este algoritmo se presenta en el apéndice 5.

### Evaluación del desempeño predictivo del modelo

Se evaluó el modelo desarrollado por medio de parámetros *(metrics)* como la exactitud, la sensibilidad y la especificidad. Cada uno de estos parámetros se describe en el apéndice 6.

### Evaluación del comportamiento de las variables incluidas

Se evaluó el modelo desarrollado usando iteraciones simuladas para analizar el comportamiento de las variables involucradas. Los signos y síntomas de la diabetes representan conceptos dinámicos que evolucionan constantemente en el tiempo, mediante la interacción entre ellos ^11^. Para hacer las simulaciones, se denominó vector inicial al conjunto de conceptos o variables de cada paciente en un momento inicial. Este vector fue ingresado al modelo, el cual en cada iteración se multiplicó con la matriz W y, de esta manera, se generaron activaciones de conceptos o variables a lo largo del tiempo. Una activación puede ser vista como la aparición de un síntoma después de una iteración determinada. Tras el proceso de inferencia, se obtiene un vector final que indica el estado en que los signos y síntomas ya no evolucionan más ^17^.

### Consideraciones éticas

Esta investigación se considera un estudio sin riesgo, según la Resolución 8430 de 1993 ^20^, debido a que no se utilizó ningún recurso vivo, agentes o muestras biológicas, datos personales, entrevistas o encuestas. Además, no representa ningún riesgo para la vida, el ambiente o los derechos humanos.

## Resultados

Se llevó a cabo un análisis descriptivo de las variables almacenadas en el conjunto de datos. En el cuadro 1, se muestra la distribución de frecuencias absolutas y relativas para las características con respecto a la diabetes. Con relación al sexo, se observa que la frecuencia de la diabetes es mayor en mujeres (33,2 %); para el caso de la población no diabética, los hombres se encontraron con mayor frecuencia (34,8 %). La edad fue la única variable numérica del conjunto de datos; los individuos tenían edades comprendidas entre los 16 y los 90 años, con un promedio de 48 años y una desviación estándar de 12 años. Los pacientes con diabetes presentaron un mayor promedio de edad (media = 49,1 ± 12,1) que los pacientes sin diabetes (media = 46,4 ± 12,1). Con relación a las variables clínicas, se encontró que la poliuria, la polidipsia, la pérdida repentina de peso, la debilidad, la polifagia, la visión borrosa y la parálisis parcial, se presentaron con mayor frecuencia en los individuos con diabetes.

**Cuadro 1:** Distribución de frecuencias absolutas y relativas de las características presentes en el conjunto de datos

Característica	Categoría	n (%)	
		No diabético	Diabético
Sexo	Femenino	19 (3,7)	173 (33,2)
	Masculino	181 (34,8)	147 (28,3)
Poliuria	Ausencia	185 (35,6)	77 (14,8)
	Presencia	15 (2,9)	243 (46,7)
Polidipsia	Ausencia	192 (36,9)	95 (18,3)
	Presencia	8 (1,5)	225 (43,3)
Pérdida repentina de peso	Ausencia	171 (32,9)	132 (25,3)
	Presencia	29 (5,6)	188 (36,2)
Debilidad	Ausencia	113 (21,7)	102 (19,3)
	Presencia	87 (17,7)	218 (41,3)
Polifagia	Ausencia	152 (29,2)	131 (25,2)
	Presencia	48 (9,2)	189 (36,4)
Candidiasis genital	Ausencia	167 (32,1)	237 (45,6)
	Presencia	33 (6,3)	83 (16,0)
Visión borrosa	Ausencia	142 (27,3)	145 (27,9)
	Presencia	58 (11,2)	175 (33,6)
Prurito	Ausencia	101 (19,4)	166 (31,9)
	Presencia	99 (19,1)	154 (29,6)
Irritabilidad	Ausencia	184 (35,4)	210 (40,3)
	Presencia	16 (3,1)	110 (21,2)
Retraso en la cicatrización	Ausencia	114 (21,9)	167 (32,2)
	Presencia	86 (16,5)	153 (29,4)
Parálisis parcial	Ausencia	168 (32,3)	128 (24,6)
	Presencia	32 (6,2)	192 (36,9)
Rigidez muscular	Ausencia	140 (26,9)	185 (35,6)
	Presencia	60 (11,5)	135 (26,0)
Alopecia	Ausencia	99 (19,0)	242 (46,5)
	Presencia	101 (19,5)	78 (15,0)
Obesidad	Ausencia	173 (33,3)	259 (49,8)
	Presencia	27 (5,2)	61 (11,7)

### Desempeño predictivo del modelo basado en mapas cognitivos difusos

Se desarrolló un modelo de mapa cognitivo difuso para la predicción de la diabetes, utilizando información sociodemográfica y clínica. En los resultados de los parámetros de evaluación, el modelo obtuvo un gran rendimiento con una exactitud del 95 %, una sensibilidad del 96 % y una especificidad del 94 %, con los siguientes hiperparámetros: *Initial population = 200; activation function = sigmoid; inference function = modified-Kosko.*

Debido a su simplicidad, el modelo se puede visualizar; en la figura 2, se muestra una representación esquemática del mapa cognitivo difuso, donde se pueden observar las relaciones e influencias de los conceptos o variables predictoras sobre la presencia de la diabetes.

**Figura 2:**
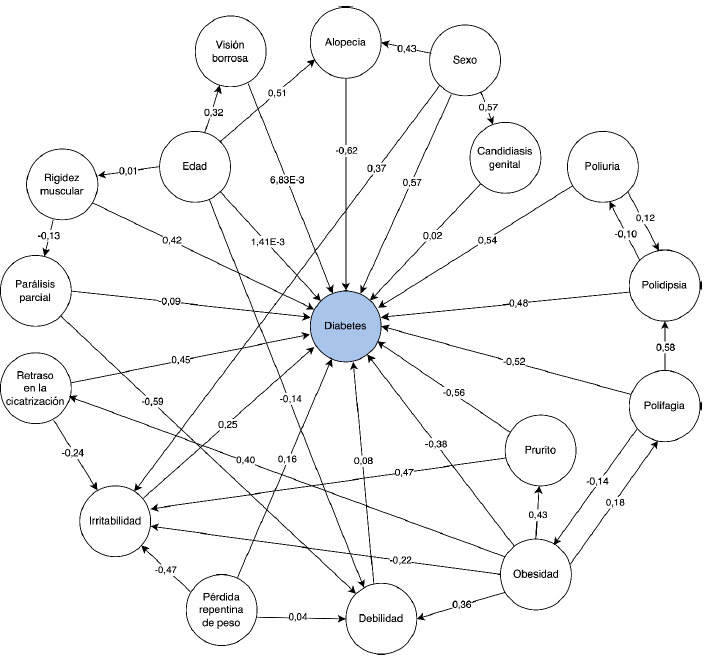
Mapa cognitivo difuso para la predicción de la diabetes

### Comportamiento de las variables usando iteraciones simuladas

**Figura 3:**
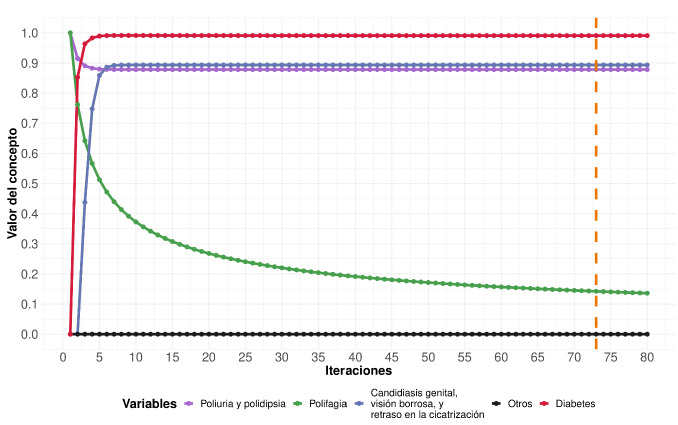
Comportamiento de las variables para la predicción de la diabetes mediante un mapa cognitivo difuso. La línea punteada anaranjada indica cuándo el sistema logra el equilibrio.

Se evaluó el modelo desarrollado usando iteraciones simuladas para analizar el comportamiento de las variables involucradas. En la figura 3, se muestra una representación esquemática de las simulaciones con mapas cognitivos difusos para diabetes. En el eje de las X de la gráfica, se muestran las iteraciones simuladas y, en el eje de las Y, se muestra el valor de las variables o conceptos. La simulación de esta figura corresponde a un paciente con poliuria, polidipsia y polifagia. Luego de varias iteraciones, el sistema logra un estado de equilibrio que indica que los conceptos no cambian de valor después de la iteración 72 (línea punteada anaranjada). En la figura 3, se puede observar cómo el modelo activa variables que no se encontraban presentes desde el inicio, como la candidiasis genital, la visión borrosa y el retraso en la cicatrización (todas estas variables representadas por la curva de color azul).

Por otra parte, se ve que el concepto relacionado con el diagnóstico de la diabetes (curva roja) es activado desde la primera iteración, lo que indica que los síntomas alertan de manera temprana sobre la presencia de la enfermedad.

## Discusión

En las últimas décadas, la diabetes ha incrementado su prevalencia y se ha convertido en una causa importante de morbilidad y mortalidad a nivel mundial ^21^. La importancia de este trabajo radica en la generación de un modelo de inteligencia artificial para la detección temprana de la diabetes que, no solo predice la enfermedad, sino que, también, permite el análisis del comportamiento de los principales factores de riesgo.

### Análisis del rendimiento e interpretabilidad del modelo desarrollado

En el presente estudio, se construyó un modelo de mapa cognitivo difuso, calibrado con el algoritmo PSO. Los resultados del modelo demostraron un rendimiento significativo, con una exactitud del 95 %, una sensibilidad del 96 % y una especificidad del 94 %. Este excelente rendimiento puede deberse, en principio, a la naturaleza misma de las variables con las que se construyó el modelo, dado que son variables críticas o determinantes que hacen parte del razonamiento clínico en el diagnóstico de diabetes. Además, la literatura científica ha demostrado que la normalización y el equilibrio de clases ^13^ podrían mejorar significativamente el rendimiento del modelo en comparación con enfoques sin preprocesamiento ^11^^,^^22^.

Los resultados del análisis del comportamiento de las variables mostraron la activación de variables como candidiasis genital, visión borrosa y retraso en la cicatrización. Los altos niveles de azúcar en sangre en individuos con diabetes no controlada pueden debilitar el sistema inmunitario, haciéndoles más propensos a las infecciones ^23^. La candidiasis genital es causada por un crecimiento excesivo del hongo *Candida* spp., y la respuesta inmunológica comprometida en la diabetes puede aumentar la vulnerabilidad ante este tipo de infección ^24^.

Los niveles elevados y prolongados de azúcar en sangre pueden dañar los pequeños vasos sanguíneos de la retina, una afección conocida como retinopatía diabética. Este daño puede provocar el debilitamiento de las paredes de los vasos y la filtración de líquidos y sangre en el ojo, lo que conduce a visión borrosa ^25^.

La hiperglucemia crónica puede provocar aterosclerosis, reduciendo el flujo sanguíneo a diversas partes del cuerpo, incluidas las heridas ^26^. Un riego sanguíneo inadecuado en el lugar de la herida significa que llegan menos nutrientes y células inmunitarias a la zona, lo que ralentiza el proceso de cicatrización ^23^^,^^27^.

Con base en los resultados del presente estudio, se comprobaron las bondades del modelo basado en mapas cognitivos difusos, pues, además de predecir la enfermedad con una buena exactitud, se puede utilizar para evaluar el comportamiento entre las variables predictoras y la presencia de diabetes. Una ventaja adicional de este tipo de modelos es que permite la representación gráfica de las iteraciones simuladas. Esto ayuda a tener una visión general de la situación clínica del paciente, para optimizar las acciones y recomendaciones con el fin de disminuir tasas de morbilidad y mortalidad.

### Comparación cualitativa con trabajos previos

En el cuadro 2, se muestra la comparación cualitativa con estudios previos, debido a que no hay estudios que utilicen el mismo conjunto de datos para realizar una comparación cuantitativa. Alam implementó un mapa cognitivo difuso intuitivo y fácil de usar, para analizar las relaciones existentes entre los factores de riesgo y los síntomas de la diabetes ^9^. El autor evaluó la relación mediante el desarrollo de seis pruebas simuladas con 10 atributos y concluyó que la edad afecta gravemente la condición diabética, seguida por el sobrepeso y la obesidad. El modelo no fue validado en un conjunto de datos y tampoco tiene la capacidad de predecir el desarrollo de la enfermedad.

**Cuadro 2:** Comparación cualitativa entre los enfoques previos de mapas cognitivos difusos y nuestro trabajo

Criterios cualitativos	Alam ^9^	Froelich y Wakulicz-Deja ^28^	Bhatia y Kumar ^10^	Nuestro trabajo
Capacidad predictiva	No	No	Sí	Sí
Evaluación del comportamiento de variables	Sí	No	No	Sí
Validación en conjunto de datos	No	Sí	Sí	Sí
Intuitivo y fácil de usar	Sí	No	Sí	Sí

Froelich y Wakulicz-Deja usaron mapas cognitivos difusos como apoyo a las decisiones médicas, con el fin de descubrir dependencias entre intervenciones médicas y los efectos en la salud generados por cambios de las condiciones de los pacientes diabéticos ^28^. El enfoque del modelo es prescriptivo, con el fin de recomendar acciones para ajustar la dosis de insulina; fue validado en un conjunto de datos reales y se tuvieron en cuenta las características temporales de los mapas cognitivos difusos. Una desventaja de este modelo es la capacidad de interpretación, pues los criterios definidos para las variables en estudio no son claros, ni de fácil interpretación para el personal médico.

Bhatia y Kumar desarrollaron un sistema de apoyo a la toma de decisiones médicas basado en mapas cognitivos difusos para predecir la diabetes a partir de signos y síntomas ^10^. El enfoque utilizado se probó en 50 pacientes y presentó una buena capacidad de predicción, con una exactitud del 96 %. Los autores diseñaron una herramienta de soporte flexible, de fácil uso e interpretación; sin embargo, no se analizó la relación existente entre los factores de riesgo y la presencia de diabetes para definir las variables más influyentes sobre el desarrollo de la enfermedad.

A diferencia de los enfoques reportados en la literatura científica, en este artículo desarrollamos y proponemos un modelo de mapa cognitivo difuso que hemos enfocado en la predicción de la diabetes. El modelo logró una buena capacidad de predicción con una exactitud del 95 % al ser calibrado y validado en un conjunto de 640 datos. Además, nuestro modelo puede ser utilizado como un sistema de apoyo a las decisiones médicas debido a su facilidad de visualización e interpretación.

Esta investigación presenta algunas limitaciones. En primer lugar, solo se utilizó un conjunto de datos de una región específica; por tal motivo, se recomienda realizar más investigaciones utilizando conjuntos de datos más grandes, variados y contextualizados. Por otra parte, solo se usaron dieciséis variables predictoras y no otras variables de interés para el diagnóstico de la diabetes, como el ejercicio físico y los resultados de las pruebas de laboratorio. El desarrollo de modelos con este tipo de variables podría proporcionar un análisis más sólido de la diabetes.

Otra limitación es que los datos empleados para la construcción de los modelos fueron recolectados mediante una encuesta. Consideramos que la subjetividad es un factor determinante en la exactitud de la información suministrada por los pacientes. Sería interesante una recolección de datos directa con mediciones controladas en los pacientes con diabetes e individuos sin la enfermedad. Finalmente, con los datos de futuros estudios locales que permitan validar esta información, se propone el desarrollo de una herramienta *web* o *software* de fácil acceso, como sistema de apoyo para la toma de decisiones médicas en instituciones que ofertan servicios de salud para la prevención y el diagnóstico oportuno de la diabetes.

En esta investigación, se propuso un modelo de inteligencia artificial basado en mapas cognitivos difusos para la predicción de la diabetes y el análisis del comportamiento de las variables relacionadas con la enfermedad. Además, se demostraron los beneficios de usar la inteligencia artificial para construir y usar herramientas que permiten apoyar la toma de decisiones con respecto a la detección de la diabetes. Específicamente, con el modelo desarrollado no solo se detectó la diabetes con un excelente desempeño, sino que, también, permitió analizar las variables involucradas en la predicción de esta enfermedad. De esta manera, generamos una herramienta valiosa para la detección temprana de la enfermedad, que podría ayudar a disminuir las tasas de morbilidad y mortalidad.

## Archivos suplementarios

### Apéndice 1

**Cuadro 1:** Breve descripción de las variables del conjunto de datos en estudio

Concepto	Variable	Tipo de variable	Naturaleza	Breve descripción
C1	Edad	Sociodemográfica	Numérica	Tiempo que transcurre desde el nacimiento
C2	Sexo	Sociodemográfica	Binaria	Características biológicas que diferencian en femenino o masculino
C3	Poliuria	Clínica	Binaria	Aumento del volumen de orina excretada
C4	Polidipsia	Clínica	Binaria	Aumento excesivo de la sed
C5	Pérdida repentina de peso	Clínica	Binaria	Disminución involuntaria del peso corporal
C6	Debilidad	Clínica	Binaria	Reducción de la fuerza física o muscular
C7	Polifagia	Clínica	Binaria	Aumento del consumo de alimentos por exceso de hambre
C8	Candidiasis genital	Clínica	Binaria	Infección fúngica en la zona genital
C9	Visión borrosa	Clínica	Binaria	Disminución de la agudeza visual
C10	Prurito	Clínica	Binaria	Picor o irritación de la piel que induce la necesidad de rascar
C11	Irritabilidad	Clínica	Binaria	Estado emocional relacionado con temperamento explosivo o fácil enojo
C12	Retraso en la cicatrización	Clínica	Binaria	Disminución de los mecanismos naturales para la regeneración de los tejidos
C13	Parálisis parcial	Clínica	Binaria	Pérdida parcial del movimiento muscular voluntario
C14	Rigidez muscular	Clínica	Binaria	Tensión muscular, que puede acompañarse de espasmos y dolor
C15	Alopecia	Clínica	Binaria	Caída anormal del cabello
C16	Obesidad	Clínica	Binaria	Acumulación excesiva de grasa corporal
C17	Clase	Diagnóstico	Binaria	Presencia o ausencia de diabetes

**Cuadro 2:** Representación de un mapa cognitivo difuso mediante una matriz de siete conceptos y sus relaciones

	C_1_	C_2_	C_3_	c_4_	C_5_	C_6_	C_7_
C_1_	0	0	0	0	0	0	W_17_
C_2_	W_21_	0	W_23_	0	0	0	W_27_
C_3_	0	0	0	0	W_35_	0	0
C_4_	0	0	W_43_	0	W_45_	0	0
C_5_	0	0	0	0	0		0
C_6_	0	0	0	0	0	0	W_67_
C_7_	0	0	W_73_	0	0	W_76_	0

**Cuadro 3:** Hiperparámetros para el modelo de aprendizaje mapas cognitivos difusos

Técnica	Hiperparámetros	Opciones
Mapa cognitivo difuso	Población inicial	50, 100 ,150 ,200, 250, 300
	Función de activación	Sigmoide, tanh
	Función de inferencia	Kosko-estándar, Kosko-modificado, rescaled

## Apéndice 2.

Normalización min-máx

Se utilizó la normalización mín-máx para la edad, que fue la única variable numérica del conjunto de datos. Este procedimiento ayudó a mantener las variables en el mismo rango y, también, a optimizar el tiempo en la calibración de los modelos. Con la siguiente ecuación, se expresa la normalización mín-máx:




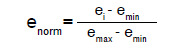




En donde: (e_norm_) es la edad normalizada, (e_i_) es la edad del individuo, (e_min_) es la edad mínima y (e_max_) es la edad máxima.

## Apéndice 3.

Explicación de un mapa cognitivo difuso

En la figura 2 (apéndice 4), se observa una estructura gráfica con siete nodos que representa un mapa cognitivo difuso simple. Los nodos son los conceptos (C) y la relación entre cada concepto (influencias) está representada por un peso (W), que se expresa por medio de una flecha dirigida desde un nodo de origen a un nodo de destino. El subíndice en la W de cada flecha indica la dirección de esa relación, es decir, W_17_ representa la relación entre el concepto 1 (C_1_) y el concepto 7 (C_7_). En el ámbito clínico, los nodos o conceptos suelen ser, por ejemplo, factores de riesgo asociados con una enfermedad, los síntomas de una enfermedad o las pruebas de laboratorio ^1^.

Otra forma de representar las relaciones entre los conceptos del sistema en estudio es mediante una matriz cuadrada, que almacena la información de las influencias o pesos entre los conceptos de un mapa cognitivo difuso ^2^. Como ejemplo, en el cuadro 2 del apéndice 1, se representa la matriz de pesos para el mapa cognitivo difuso de la figura 2 del apéndice 4.

## Apéndice 4.

Figuras adicionales

**Figura 1:**
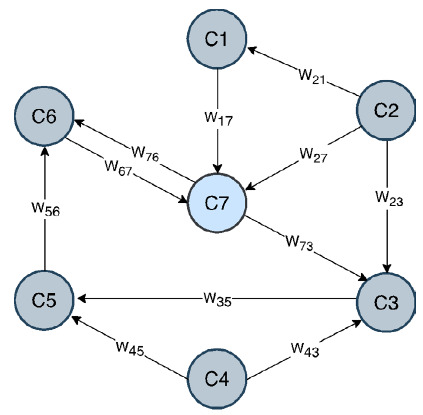
Representación gráfica de un mapa cognitivo difuso simple, con siete conceptos (nodos) y flechas que indican las relaciones entre ellos

**Figura 2:**
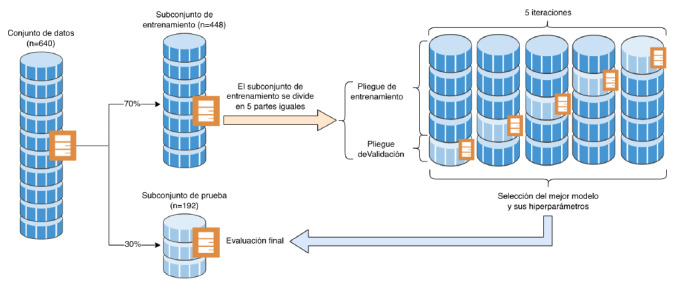
Esquema general de la validación cruzada de cinco repeticiones

## Apéndice 5.

Descripción del algoritmo *Particle Swarm Optimization* (PSO)

La optimización de los mapas cognitivos difusos con el algoritmo PSO (optimización por enjambre de partículas) se logra mediante el empleo de dos ecuaciones ^3^. La primera de ellas permite modelar la actualización de la velocidad de la partícula (en nuestro caso, la partícula es el mapa cognitivo difuso):




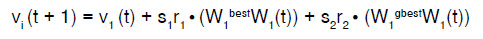




En donde: v_i_ corresponde a la velocidad de la partícula; es el coeficiente cognitivo, mediante el cual la partícula i busca ubicarse en la posición donde ha obtenido resultados más exitosos; s_2_ es el componente social (comportamiento colectivo), por medio del cual la partícula i busca alinearse a la mejor posición hallada por el enjambre hasta el momento; por su parte, r_1_ y r_2_ corresponden a valores aleatorios que siguen una distribución uniforme; las variables W_1_^best^ y W_1_^gbest^ corresponden a la mejor posición obtenida por una partícula en concreto y a la mejor posición obtenida por cualquier partícula del enjambre, respectivamente. La siguiente ecuación (4) permite modelar la posición actualizada de la partícula i(FCM), una vez actualizadas las velocidades, según la anterior ecuación:




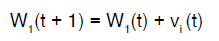




A partir de las anteriores operaciones, el algoritmo genera una matriz de pesos optimizada. El algoritmo PSO es eficaz, porque permite diseñar y entrenar modelos predictivos de mapas cognitivos difusos, empleando datos reales y artificiales, y superando los sesgos inducidos por la intervención humana durante el proceso (5).

## Apéndice 6.

Parámetros para evaluar el desempeño del modelo

El modelo desarrollado se evaluó según su capacidad para detectar la diabetes, mediante el empleo de los siguientes parámetros:

*Exactitud:* corresponde al porcentaje de ejemplos clasificados en forma correcta entre el número total de ejemplos clasificados. Entre mayor sea este porcentaje, mayor será el rendimiento total del modelo que se prueba.




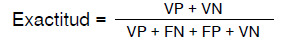




De acuerdo con la fórmula, VP representa el valor de los verdaderos positivos; VN corresponde al valor de los verdaderos negativos; FN el valor de falsos negativos y FP el valor de falsos positivos.

*Especificidad:* es la medida de la capacidad que posee el modelo para clasificar los casos negativos frente a los verdaderamente negativos.




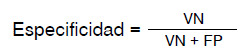




*Sensibilidad:* es la medida de la capacidad que tiene el modelo para clasificar los casos positivos frente a los verdaderamente positivos.




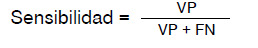




### Referencias

1. Hoyos W, Aguilar J, Toro M. PRV-FCM: An extension of fuzzy cognitive maps for prescriptive modeling. Expert Syst Appl. 2023;23:1-15. https://doi.org/10.1016/j.eswa.2023.120729

2. Hoyos W, Aguilar J, Toro M. A clinical decision-support system for dengue based on fuzzy cognitive maps. Health Care Manag Sci. 2022;25:666-81. https://doi.org/10.1007/s10729-022-09611-6

3. Kennedy J, Eberhart, R. Particle swarm optimization. Proceedings of ICNN'95 - International Conference on Neural Networks. Perth, WA, Australia, 1995, p. 1942-48. vol. 4. https://doi.org/10.1109/ICNN.1995.488968

4. Salmeron JL, Rahimi SA, Navali AM, Sadeghpour A. Medical diagnosis of rheumatoid arthritis using data driven PSO-FCM with scarce datasets. Neurocomputing. 2017;232:104-112. https://doi.org/10.1016/j.neucom.2016.09.113

5. Hoyos W, Aguilar J, Toro M. Federated learning approaches for fuzzy cognitive maps to support clinical decision-making in dengue. Eng Appl Artif Intell. 2023;123:1-15. https://doi.org/10.1016/j.engappai.2023.106371
